# Long-Term Cognitive Performance of Retired Athletes with Sport-Related Concussion: A Systematic Review and Meta-Analysis

**DOI:** 10.3390/brainsci9080199

**Published:** 2019-08-13

**Authors:** Yanjie Zhang, Yongzhi Ma, Shihui Chen, Xiaolei Liu, Hye Jung Kang, Siera Nelson, Samantha Bell

**Affiliations:** 1 Chinese Traditional Regimen Exercise Intervention Research Center, Beijing Sport University, Beijing 100084, China; 2 Health and Exercise Science Laboratory, Institute of Sports Science, Seoul National University, Seoul 08826, Korea; 3 Division of Sports Science and physical education, Tsinghua University, Beijing 100084, China; 4 Department of Kinesiology, Texas A&M University Texarkana, Texarkana, TX 75503, USA

**Keywords:** sport-related concussion, cognitive function, retired athletes, synthesized analysis

## Abstract

*Objective:* The purpose of this systematic review is to quantitatively estimate (or invest) the impacts of sports-related concussions (SRCs) on cognitive performance among retired athletes more than 10 years after retirement. *Methods:* Six databases including (MEDLINE, Scopus, Web of Science, SPORTDiscus, CINAHL, and PsycArtilces) were employed to retrieve the related studies. Studies that evaluate the association between cognitive function and the SRC of retired athletes sustaining more than 10 years were included. *Results:* A total of 11 studies that included 792 participants (534 retired athletes with SRC) were identified. The results indicated that the retired athletes with SRCs, compared to the non-concussion group, had significant cognitive deficits in verbal memory (SMD = −0.29, 95% CI −0.59 to −0.02, I^2^ = 52.8%), delayed recall (SMD = −0.30, 95% CI –0.46 to 0.07, I^2^ = 27.9%), and attention (SMD = −0.33, 95% CI −0.59 to −0.06, I^2^ = 0%). Additionally, meta-regression demonstrated that the period of time between testing and the last concussion is significantly associated with reduced verbal memory (*β* = −0.03681, *p* = 0.03), and increasing age is significantly associated with the verbal memory (*β* = −0.03767, *p* = 0.01), immediate recall (*β* = −0.08684, *p* = 0.02), and delay recall (*β* = −0.07432, *p* = 0.02). *Conclusion:* The retired athletes who suffered from SRCs during their playing career had declined cognitive performance in partial domains (immediate recall, visuospatial ability, and reaction time) later in life.

## 1. Introduction

Recently, considerable attention has been focused on the issue of sports-related concussions (SRCs), which is becoming a significant public health problem. An SRC is a subset of mild traumatic brain injury (TBI), which is commonly caused by a direct blow to the head, face, or elsewhere on the body with a strong external force [1]. According to the research, sports-related concussions cause a range of clinical symptoms, for example, headache, cognitive, or emotional fluctuation [2,3]. Early works have shown that the injury rate of SRC is increasing year by year, especially in adolescents [4,5]. Moreover, the statistical results from the Centers for Diseases Control and Prevention (CDC) showed that the contact sports such as football, hockey, lacrosse, and rugby are the most common form of concussion, and the number of sports-related concussions occurring in the United States has reached 3.8 million annually [6]. The direct or indirect cost of concussion-related medical care is approximately $60 billion a year in the United States [7]. 

There has been an emergence of interest in trying to understand the impacts of athletes following a SRC. Previous studies documented that athletes who suffered from SRCs were more likely to report poorly in cognitive performance during an acute stage [8,9,10,11,12]. For example, Master et al. examined the effects of SRC on cognitive function (e.g., verbal/visual memory, attention, and visuoperceptual ability) in professional soccer players [13]. The study showed that cognitive impairment was attributed to the cumulative concussions. A review study on the negative impacts of SCRs reported that repeated concussions decreased functions and capabilities in multiple cognitive domains; however, cognitive recovery was observed within two weeks [14]. Therefore, the limited duration of the post-SRC and the rapid cognitive recovery may have allowed most researchers to ignore the effects of long-term concussions.

Nevertheless, conclusions about the relationship between long-term cognition and the SRCs are unclear among senior retired athletes. As the frequency of concussions increase, the deficits in learning and memory, processing speed, and verbal fluency presented an aggravation trend in university-level football and ice hockey players after one single season [15]. More recent evidence suggested that deficits may remain in partial cognitive domains for two to three years after a SRC [16,17], and even continue to cause neuropsychological dysfunction later in life [18,19]. One recent cross-sectional study including 93 former collegiate players was conducted by Montenigro et al., showing that there were close relationships between SRCs and later-life cognitive consequences [20]. Retired football athletes with SRCs had poor cognition in memory, and the study also found that relative to normal adults, National Football League (NFL) players suffering from SRCs were more likely to develop mild cognitive impairment [18]. It is possible to explore whether SRCs contribute to neurodegeneration through examining a postmortem diagnosis of traumatic brain injury [21]. Conversely, findings from other studies are not consistent with the former points. For example, recent evidence suggested that a SRC was not a key factor in declining cognition through analyzing 35 retired NFL players over the age of 50 years [22]. It appears that SRCs are only associated with disorders of the brain nervous system, rather than neurological performance [23]. 

Given that there are inconsistencies across individual studies, it is necessary to conduct a systematic study on this controversial issue to provide strong evidence that identifies the risk effects of SRCs on long-term cognition. The present study used a systematic review and meta-analysis to reveal the essential association between cognitive function later in life and the retired athletes with a history of SRC during competitions, and examine whether the number of concussions, the period of time since the last concussion, career years, and age had different effects on cognitive performance.

## 2. Methods

### 2.1. Search Strategy

Six electronic databases including MEDLINE, Scopus, Web of Science, SPORTDiscus, CINAHL, and PsycArtilces from inception to April 2019 were searched to retrieve the relevant articles. According to searching strategy in the previous study [24], the combination of three groups of key terms were conducted as follows: (i) brain concussion OR mild traumatic brain injury OR concussion OR sport* related concussion* (* are used to expand our search by including different word endings and spellings) OR craniocerebral trauma; (ii) retired player OR retired athletes OR former player OR former athlete; (iii) cognition OR cognitive function OR brain function OR memory OR executive function. The review authors subsequently retrieved the bibliography of included studies to ensure the eligible articles. 

### 2.2. Inclusion and Exclusion Criteria 

Studies were included for further analysis if they met the following criteria: (i) participants were high school, university, or professional players who suffered a sport-related concussion, and they were confirmed by sports physicians or doctors according to the practice parameters on concussion; (ii) participants in the concussed group had concussions compared to the control group (no concussed participants); (iii) the evaluation time was more than 10 years since the last concussion; (iv) at least one cognitive outcome (e.g., memory, attention, executive function) was present to calculate the effect size (ES) reported in the original study. The studies were excluded if they: (i) were published in non-English journals; (ii) used other methods (e.g., electroencephalography) to assess the cognitive performance; or (iii) were abstracts, animal studies, case studies, review publications, commentaries, or editorials. 

### 2.3. Data Extraction

Following a predefined strategy, two authors independently screened the initial titles and abstracts. Subsequently, potential relevant full-text articles were retrieved for further examination. One author extracted the results (author, year of publication, characteristics of participants, sample size, number of concussion times, and cognitive measurement tools and outcomes) from each study, and a second author corroborated the first. Disagreements between authors during extraction were adjudicated to reach a consensus by a third reviewer.

### 2.4. Assessment of Risk of Bias 

The risk of bias of each included study was independently assessed by two reviewers using the modified Downs and Black checklist [25], which provides a critical profile to evaluate the strengths and weaknesses of study methodology. It comprises 21 items in five domains: reporting (nine items), external validity (three items), internal validity bias (five items), internal validity confounding selection bias (four items), and power (one item). The evaluable scores are >14, 10–13, and ≤9, representing strong quality, moderate quality, and limited quality, respectively [25].

### 2.5. Synthesized Analysis

All data were analyzed in Comprehensive Meta-Analysis Software. The standardized mean difference (SMD) as ES estimate was calculated in a random effects model, which could incorporate the variability between studies. The ES of each study was calculated by dividing the difference between the control group and concussed group by their pooled standard deviation, and the positive ES represented the better performance in the control group. Usually, the magnitude of ES is defined as: small (0.2–0.49), moderate (0.50–0.79), and large (≥0.8) [26]. Further, heterogeneity between studies was examined using the Q statistic and I^2^ statistic, which has been classified as: I^2^ <25% (low heterogeneity), I^2^ <50% (moderate heterogeneity), and I^2^ >75% (high heterogeneity), respectively [27]. Publication bias was examined in Egger’s test and was determined from a corresponding p < 0.05. Notably, potential variances (number of concussion times, times since last concussion, career years, and participant age) may affect the results; therefore, further moderator analyses were performed. Herein, for the number of concussion times, we coded two or more concussions as multiple concussions. If a mean number ranging from 1 to 2 was given, incidents of multiple concussions were coded when the proportion of one concussion in a concussed group was more than 70%. 

## 3. Results

### 3.1. Search Results

**Figure 1:**
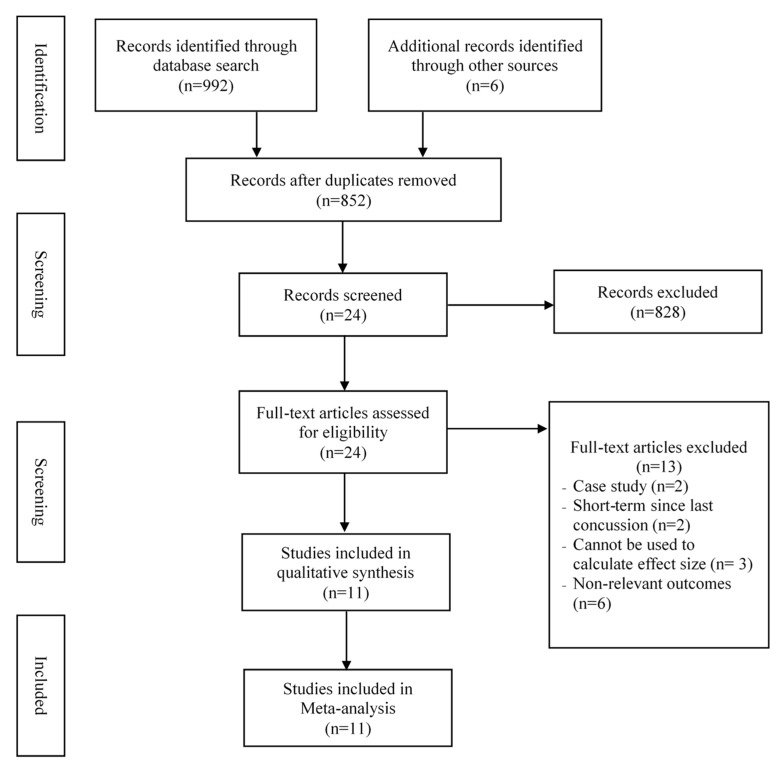
Flowchart of the study selection.

Figure 1 depicts the detailed flow of the study selection. The initial database search yielded 998 articles. After removing duplications (*n* = 146), 852 records were screened through identifying the titles and abstracts, and 24 records were retrieved for full-text reviews. Finally, a total of 11 studies [28,29,30,31,32,33,34,35,36,37,38] that met the inclusive criteria were included for this meta-analysis. 

### 3.2. Characteristics and Risk of Bias of Included Studies

Table 1 summarizes the main characteristics of included studies. The included studies were published between 2009–2019, comprising 792 participants (concussed: 534, control: 258) from high school and college competitions, with a mean age range from 48 to 68 years. The included studies were almost entirely evaluating males (8/11 studies were male in recruitment). The participants in the concussed group and control group for each study had similar levels of education (around 16 years). All the concussed participants were self-reported concussion and sports physicians or certified sports trainers. Nine out of 11 studies were done on athletes competing in full contact sports (football and rugby) that have numerous concussive impacts during competition. The time since the last concussion ranged from 15 to 37 years.

With regard to the methods for measuring, cognitive performance for retired athletes was measured using a wide variety of instruments in the included studies, such as the Immediate Post-Concussion Assessment and Cognitive Test, Rey–Osterrieth Complex Figure Test, Symbol Digit Test, and Judgment of Line Orientation Test (Table 1). To explain the various cognitive functions, the measurements were categorized to five domains: immediate recall, delay recall, visual ability, reaction time, attention, and verbal memory 

The scores for risk of bias of the included studies are shown in Table 1. All the included studies were 11 points out of a total score of 22, representing a moderate quality.

### 3.3. Meta-Analysis of Cognitive Performance

Table 2 presents the synthesized results on different domains. The pooled results showed that the concussed group had a significant deficit in verbal memory (SMD = −0.29, 95% CI −0.59 to −0.02, I2 = 52.8%, *p* = 0.03), delay recall (SMD = −0.30, 95% CI −0.55 to −0.05, I2= 24.1%, *p* = 0.02), and attention (SMD = −0.33, 95% CI −0.59 to −0.06, I2 = 0%, *p* = 0.02) compared with the control group. In term of other domains (immediate recall (SMD = −0.20, 95% CI −0.46 to 0.07, I2= 27.9%, *p* = 0.15), visuospatial ability (SMD = −0.11, 95% CI −0.28 to 0.05, I2= 12.5%, *p* = 0.18), and reaction time (SMD = −0.08, 95% CI −0.40 to 0.23, I2= 64.5%, *p* = 0.60)), there were no significant differences between the concussed group and control group. 

### 3.4. Moderator Analysis

To examine the impacts of potential variances, the moderator analysis was performed subsequently based on the time since the last concussion, the number of times that concussions occurred in their athletic career, career years, and participant age (Table 3). The meta-regression results revealed that the increasing time between testing and the last concussion was a significant factor in influencing verbal memory (β = −0.03681, Q = 4.80, df = 1, *p* = 0.03), and the participant age was also a significant factor in influencing verbal memory (β = −0.03767, Q = 7.26, df = 1, *p* = 0.01), immediate recall (β = −0.08684, Q = 5.37, df = 1, *p* = 0.02), and delay recall (β = −0.07432, Q = 5.48, df = 1, *p* = 0.02). However, there were no significant associations between cognitive outcomes and number of concussion and career years (all *p* > 0.05).

## 4. Discussion

SRCs are common among currently competing collegiate and professional athletes, yet underreported in the literature and equivocal at best when attempting to correlate impaired cognition later in life with an SRC after the athlete has retired. This review study may be the first to evaluate this relation through identifying 11 relevant cross-sectional studies worldwide. Our major findings were that the retired male athletes with a self-reported history of SRCs may have poor cognition in partial domains, especially for verbal memory, delay recall, and attention, and the issue of changes in reaction time can not be concluded at present because of the insufficient studies. 

Cognitive function consisting of various domains (e.g., immediate memory, delayed recall, and attention) was the most frequently measured factor in concussion research. According to the measurement of each study, the present study categorized the similar cognitive tests into specific domains, rather than more general constructs such as memory, which may minimize the influence on the size of ESs [39]. With regard to memory, the aggregated result from this meta-analysis revealed that the retired athletes with a history of SRC demonstrated significant memory loss in both verbal memory and delayed recall scores compared with non-concussed athletes. These significant effects were consistent with a previous meta-analysis, which suggested that reduced delayed recall with small effect was attributed to multiple concussions [40]. It is likely that older retired football players had a hippocampal volume loss versus adults who never experienced SRC because the hippocampus, which is located in the middle cranial fossa, is in a position that is more prone to injury [41]. Furthermore, regression analyses indicated that an increase in the mean age (48 to 68 years) of retired athletes corresponded to a significant decline in their memory (verbal memory, immediate recall, and delay recall), and that verbal memory score also showed a decline following the increasing time between testing and the last concussion. These results were in line with a recent study that provided evidence that the increasing age and the increasing time since the last concussion may synergistically result in the development of certain cognitive deficits for athletes with a history of SRC [36]. 

In terms of attention, we observed a small negative ES (–0.33), implying that the retired athletes with SRCs during their playing careers had significant deficits. It is probable that they suffered multiple SRC or SRC injuries that worsened their neuropsychological performance in the attention domain. In addition, when a concussion occurs, the neural network and its mechanisms are disrupted, which lead to decreased attention ability, and more mechanisms are needed to compensate for the damaged brain [5]. However, due to the lack of research that we found, we did not conduct a regression analysis on attention.

In spite of a growing body of studies on cognition and concussion in retired players, there is a lack of effective evaluation of the role of SRCs. Through widely searching the relevant studies, to our knowledge, this meta-analysis is the first to associate the cognitive performance of retired athletes with a history of SRC (sustained 15 to 35 years prior) to impairment years later. However, it should be noted that some limitations exist in this meta-analysis. First, only peer-reviewed articles in English were included for our meta-analysis, excluding other types of papers and language articles. To some extent, these standards may lead to publication bias. Second, self-reported history of concussion was commonly used in original studies, which creates the possibility of memory error—for example, correctly remembering the number of concussions during their career—leading to inaccurate results. Besides, the included studies did not report the levels of SRCs and also tabulate recovery time from SRCs. Third, the participant information in each study was variable, such as current levels of physical activity, which may be a confounder in cognitive testing. Four, there may be bias in participant selection, because it is not clear whether the selected participants represented the whole retired athlete population with a career history of SRC. In future research, scientists should focus on a representative group of SRC athletes. A prospective longitudinal study was used to associate cognitive performance with SRC during their playing days, and the objective variations corresponding to each SRC of the athletes were recorded by brain imaging to support further evaluation. 

## 5. Conclusions

To sum up, it is reasonable to evaluate the cognition ability of retired athletes by retrospective studies. This meta-analysis indicates that there is an association between males who participated in professional and highly competitive contact sports (such as American football and rugby) and self-reported concussions during their play in verbal memory, delay recall and attention 10 years after retirement from the sport. However, this conclusion should be interpreted with caution, given the wide spread in inter-group heterogeneity and no control over possible confounders. 

## Figures and Tables

**Table 1:** Characteristics of randomized controlled trials in the meta-analysis.

Study/Year	Participants				Sports Type and Level; Career Years	Time Since Last Concussion (Mean Year)	Cognitive Outcomes (Instrument)	Downs and Black Score; Quality
	Reported Concussion	Mean Age (Concussed/Control)	Sample Size (Concussed/Control) n Times of Concussion Male (%)	Year of Education				
De Beaumont et al. (2009) [28]	Self-report and sports physician confirmed	61; 59	19; 21 1–5 n.r	18; 18	Mix; C n.r	34.74	Immediate recall(RCFT) Delayed recall (RCFT) Visual ability (RCFT)	11, moderate
Hume et al. (2017) [29]	Self-report and doctor confirmed	41.3; 44.9; 42.1	103; 198; 65 1+ 100%	n.r	Rugby; P, C 23	25	Verbal memory (CNS-VS) Attention (CNS-VS) Reaction time (CNS-VS) Visual ability (CNS-VS)	11, moderate
Mcmillan et al. (2016) [30]	Self-report and doctor confirmed	53; 55	52; 29 14 100%	16; 17.3	Rugby; P 22.4	20	Immediate recall (RAVLT) Delayed recall (RAVLT) Reaction time (SART) Attention (SDT) Visual ability (LOT )	11, moderate
Misquitta et al. (2018) [31]	Self-report and sports physician confirmed	55.6; 50.8	53; 25 4 100%	16; 16	Football; P 9	15	Immediate recall (RAVLT) Delayed recall (RAVLT) Verbal memory (RVDLT)	11, moderate
Multani et al. (2016) [32]	Self-report and certified athletic trainer confirmed	49.6; 46.7	18; 17 5.4 100%	17.3; 16.4	Football; P 7.8	16.2	Immediate recall (RAVLT) Delayed recall (RAVLT)	11, moderate
Pearce et al. (2018) [33]	Self-report and sports physician confirmed	48.4; 48.8	25; 25 8.5 100%	13.9; 14.8	Rugby; P n.r	18.8	Verbal memory (CNT) Visual ability (CNT) Reaction time (CNT)	11, moderate
Ruiter et al. (2019) [34]	Self-report and certified athletic trainer confirmed	57.6; 53.7	19; 20 4 n.r	16.7; 16	Football; P 7.8	28	Verbal Memory (ImPACT) Visual Memory (ImPACT) Attention (ImPACT) Reaction Time (ImPACT)	11, moderate
Strain et al. (2015) [35]	Self-report and sports physician confirmed	68; 59	20; 21 3.8 n.	16.5; 15.9	Football; P 8.9	20	Verbal memory (CVLT)	11, moderate
Tarazi et al. (2018) [36]	Self-report and sports physician confirmed	53.4; 50	45; 25 5.07 100%	16.13; 16.12	Football; P 7.9	17.33	Verbal memory (RAVLT) Immediate recall (RAVLT) Delayed recall (RAVLT) Attention (Trial B)	11, moderate
Terry et al. (2018) [37]	Self-report and certified athletic trainer confirmed	53.1; 50	20; 20 4.3 100%	15.7; 15.3	Football; H n,r	36.7	Immediate recall (RBANS) Delayed recall (RBANS) Verbal memory (RBANS) Attention (RBANS) Visual ability (RBANS)	11, moderate
Tremblay et al. (2013) [38]	Self-report and sports physician confirmed	60.9; 58.13	15; 15 2.08 100%	16.67; 17.27	Mix; C n.r	24	Verbal memory (RAVLT) Visual ability (TCFT) Attention (SDT)	11, moderate

C: Collegiate player; H: High school player; P: Professional player; CNS–VS: CNS Vital Signs; CNT Cambridge Neuropsychological Test Automated Battery; CVLT: California Verbal Learning Test; ImPACT: Immediate Post–Concussion Assessment and Cognitive Test; LOT: Judgment of Line Orientation Test; n.r: not reported; RAVLT: Rey Auditory Verbal Learning Test; RVDLT: Rey Visual Design Learning Test; RBANS: Repeatable Battery for the Assessment of Neuropsychological Status; RCFT: Rey–Osterrieth Complex Figure Test; SART: Sustained Attention to Response Task; SDT: Symbol Digit Test; TCFT: Taylor complex figure test.

**Table 2:** Synthesized results for the effects of concussed group vs. control group.

Outcomes	Number of Trials	SMD	95% CI	*I^2^* %	Between-Group Heterogeneity			Publication Bias
					*Q*–Value	*df*(*Q*)	*p*–Value	Egger’s Test (*p*)
Verbal memory	6	−0.29 *	−0.59 to −0.02	52.8%	10.59	5	0.06	0.05
Immediate recall	6	−0.20	−0.46 to 0.07	27.9%	6.94	5	0.23	0.24
Delay recall	7	−0.30 *	−0.55 to −0.05	24.1%	7.90	6	0.25	0.68
Visuospatial ability	9	−0.11	−0.28 to 0.05	12.5%	9.14	8	0.33	0.68
Reaction time	5	−0.08	−0.40 to 0.23	64.5%	11.25	4	0.02	0.08
Attention	4	−0.33 *	−0.59 to −0.06	0%	2.36	3	0.50	0.94

* *p* < 0.05; SMD = Standardized mean difference.

**Table 3:** Moderator analysis for the effects of concussed group vs. control group.

Outcomes	Continuous Predictors	Number of Trials	*β*	95% CI	*Q*–Value	df(*Q*)	*p*–Value
Verbal memory	Time between testing and last concussion	6	–0.03681	–0.06974 to –0.00387	4.80	1	0.03 *
	n times of concussion	6	–0.04266	–0.08715 to 0.00182	3.53	1	0.06
	Career years	5	0.02435	–0.00204 to 0.05075	3.27	1	0.07
	Age	6	–0.03767	–0.06506 to –0.01027	7.26	1	0.01 *
Immediate recall	Time between testing and last concussion	6	–0.01184	–0.03596 to 0.01229	0.92	1	0.34
	n times of concussion	6	–0.01554	–0.06829 to 0.03720	0.33	1	0.56
	Career years	3	–0.02751	–0.53642 to 0.04140	2.82	1	0.09
	age	6	–0.08684	–0.16033 to –0.01336	5.37	1	0.02 *
Reaction time	Time between testing and last concussion	5	–0.04295	–0.10872 to 0.02282	1.64	1	0.20
	n times of concussion	5	–0.03419	–0.07068 to 0.00230	3.37	1	0.07
	Career years	4	–0.02752	–0.07109 to 0.01604	1.53	1	0.22
	Age	5	0.03489	–0.00052 to 0.07030	3.73	1	0.053
Delay recall	Time between testing and last concussion	7	–0.01113	–0.03488 to 0.01262	0.84	1	0.36
	n times of concussion	7	–0.03056	–0.08352 to 0.02240	1.28	1	0.26
	Career years	4	–0.03861	–0.07783 to 0.00061	3.72	1	0.054
	Age	7	–0.07432	–0.13650 to –0.01214	5.48	1	0.02 *
Visual spatial recall	Time between testing and last concussion	9	–0.00021	–0.02251 to 0.02209	0.00	1	0.98
	n times of concussion	9	–0.00275	–0.03785 to 0.03232	0.02	1	0.88
	Career years	5	0.00684	–0.02268 to 0.03635	0.21	1	0.65
	Age	9	–0.01296	–0.03713 to 0.01121	1.10	1	0.29

* *p* < 0.05.
